# Accuracy of RNA-Seq and its dependence on sequencing depth

**DOI:** 10.1186/1471-2105-13-S13-S5

**Published:** 2012-08-24

**Authors:** Guoshuai Cai, Hua Li, Yue Lu, Xuelin Huang, Juhee Lee, Peter Müller, Yuan Ji, Shoudan Liang

**Affiliations:** 1 Department of Bioinformatics and Computational Biology, The University of Texas MD Anderson Cancer Center, Houston, Texas 77030, USA; 2 Department of Stem Cell Transplantation and Cellular Therapy, The University of Texas MD Anderson Cancer Center, Houston, Texas 77030, USA; 3 Department of Leukemia, The University of Texas MD Anderson Cancer Center, Houston, Texas 77030, USA; 4 Department of Biostatistics, The University of Texas MD Anderson Cancer Center, Houston, Texas 77030, USA; 5 Department of Mathematics, The University of Texas at Austin, Austin, Texas 78712, USA

## Abstract

**Background:**

The cost of DNA sequencing has undergone a dramatical reduction in the past decade. As a result, sequencing technologies have been increasingly applied to genomic research. RNA-Seq is becoming a common technique for surveying gene expression based on DNA sequencing. As it is not clear how increased sequencing capacity has affected measurement accuracy of mRNA, we sought to investigate that relationship.

**Result:**

We empirically evaluate the accuracy of repeated gene expression measurements using RNA-Seq. We identify library preparation steps prior to DNA sequencing as the main source of error in this process. Studying three datasets, we show that the accuracy indeed improves with the sequencing depth. However, the rate of improvement as a function of sequence reads is generally slower than predicted by the binomial distribution. We therefore used the beta-binomial distribution to model the overdispersion. The overdispersion parameters we introduced depend explicitly on the number of reads so that the resulting statistical uncertainty is consistent with the empirical data that measurement accuracy increases with the sequencing depth. The overdispersion parameters were determined by maximizing the likelihood. We shown that our modified beta-binomial model had lower false discovery rate than the binomial or the pure beta-binomial models.

**Conclusion:**

We proposed a novel form of overdispersion guaranteeing that the accuracy improves with sequencing depth. We demonstrated that the new form provides a better fit to the data.

## Background

To measure gene expression by RNA-Seq, RNA molecules are converted to DNA, sequenced, mapped to a gene database, and counted [1-3]. RNA-Seq then provides a digital readout of the gene expression levels. As the cost of next-generation sequencing drops rapidly, RNA-Seq may replace microarray methods in genome-wide surveys of gene expression. Compared to microarray technology, RNA-Seq has several advantages, including the ability to simultaneously detect mutations, discovering alternative transcript [4-6] and alternative splicing [7-10].

It is common to study the changes in gene expression under a perturbation. The perturbation can be, for example, the deletion of a gene, which is important in characterizing the function of a new gene, or it can be the stimulation of cells by a ligand, which is important in deciphering a pathway. Many experimental techniques, such as RNA interference [11], have been developed in recent years to make it easier to delete genes in mammalian cells. For an embryonic lethal gene in the mouse model, the Cre-lox system can be used to perform conditional gene knockout in a tissue-specific manner [12]. These gene deletion techniques facilitate the study of gene functions for a large fraction of mammalian genes that remain to be characterized. Furthermore, two-sample comparisons apply when studying pathways through receptor stimulation. These methods have become increasingly popular for examining signal transduction pathways holistically. In such studies, the emphasis is on the function of genes or pathways and not on the genetic background in which the study is carried out. Therefore, one repeats the experiments in the same cell line or in mice with identical genetic backgrounds, and expects to find no genetic variation. In this situation, the difference in gene expression can be due to different methods of handling the biological samples (library preparation), as well as statistical fluctuations from the finite number of tags mapped to each gene. The uncertainty in the outcome of RNA-seq in repeated experiments of identical genetic background is yet to be characterized.

Such uncertainty affects the ability to affirm which genes are differentially expressed between a sample and a control. We focus on estimating the change in gene expression because the absolute amounts of RNA, by themselves, as measured by the RPKM (reads per kilobase of read length per million mapped reads) of the sequenced tag values [2], are not useful in most cases for biological interpretation. We hypothesize that experimental uncertainty is due primarily to the library preparation steps before sequencing, that it is intrinsic to the experimental protocol, and can therefore be characterized from repeated experiments. The expression difference is estimated based on the computation of a *p-*value, which can be calculated from repeated experiments using a t-test. However, since in RNA-Seq, the expression ratio derived from low sequence reads should have a larger error, it would be valuable to statistically estimate the error due to low counts [13]. Binomial and beta-binomial [14,15] distributions can be used to characterize small tag count fluctuations.

A fundamental question in RNA-Seq analysis is how the accuracy of measured gene expression change by RNA-Seq depend on the sequencing depth [16]. Here the sequence depth means the total number of sequenced reads, which can be increased by using more lanes. A binomial distribution is often used to compare two RNA-Seq experiments. In this model, uncertainty approaches zero as  where *N* is the tag counts for the gene. Indeed, the sequencing of the same DNA in different sequencing lanes produces errors consistent with the binomial distribution [13,17]. However, comparisons of different samples have shown a dispersion larger than that given by the binomial distribution [18,19]. A beta-binomial distribution appropriately describes the overdispersion. This type of distribution has been used for the analysis of differential gene expression levels in SAGE libraries [20], and to model peptide count data with both within- and between-sample variation in label-free tandem mass spectrometry-based proteomics [21]. For the dispersion, the error is a sum of two parts: the first part goes to zero following , and the second part is a constant that is independent of *N.* The constant is ideal for describing the genetic variant. However, where genetic variations are not expected, it is inconsistent with the intuition that the accuracy of the measurement should improve with increasing depth of sequencing. In this paper, we provide empirical evidence that the error goes to zero as the tag count *N* increases, but at a slower rate than . We aim to characterize these overdispersions gene by gene, using a pair of replicate experiments. We compared the results in a dataset in which multiple replicates were also available. We used a form of overdispersion based on a beta-binomial distribution, but one in which the overdispersion parameter depends explicitly on the number of tags. The form we used was suggested during a study of the standard deviation as a function of the tag count. We demonstrated that the modified beta-binomial distribution improve performance.

## Results

### Normalization by proportion

The use of a proportion is a convenient way to compare two samples. Let *n_i_* and *m_i_* be the number of tags mapped to gene *i.* The proportion is defined as . It is convenient to use proportion because differences in proportion give rise to *p-*values using established statistics such as binomial and beta-binomial distributions. A proportion is also a convenient component of a normalization procedure.

In order to detect differential expression in two samples, we must determine the ratio of the counts in the two samples that corresponds to the same expression. One method, adapted in calculating the RPKM, assumes that the total number of tags sequenced, and equivalently the total amount of RNA, is a constant. The problem with RPKM normalization is that the number is dominated by a few genes that receive the highest sequence reads. These genes may or may not remain constant under the two experimental conditions. One could also use housekeeping genes such as POLR2A (polymeras II) or GAPDH in a normalization procedure. The problem with relying on a housekeeping gene is that the normalization depends on the choice of genes. Since the number of housekeeping genes is small, this normalization procedure is subject to fluctuation due to relatively small tag counts on these genes. Bullard et al. have shown good results with an upper-quartile normalization method [13].

**Figure 1:**
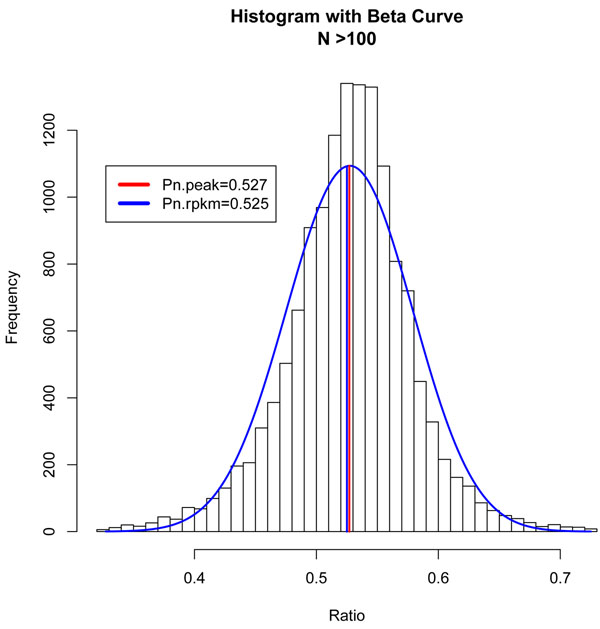
**Histogram of proportions and peak of histogram of proportion normalization**. The peak in the histogram corresponds to the largest density of genes. To determine the peak maximum, the histogram was fitted to a beta function. The blue curve shows the best fit with the maximum at *p_n_* = 0.527. This is to be compared to the proportion corresponding to RPKM normalization 0.525.

The most conservative normalization procedure assumes that the maximum number of genes remains unchanged in the two experimental conditions. This corresponds to the maximum in the histogram ratio of tag counts . The tag counts proportion *p_i_* is more convenient to use. The maximum in a histogram of *p_i_* corresponds to the neutral ratio *p_n_*, where the expression levels are assumed to be equal in the two samples. This maximum can be determined from fitting a Gaussian (or beta function) to the peak of the histogram (Figure 1). In this formulation, the RPKM normalization corresponds to choosing , where *N* and *M* are the total number of tags to genes in the experiment and control.

This peak of histogram normalization is expected to be the most reasonable procedure for the Chiang dataset [22], which consists of the wild type and knockout versions of the TDP-43 gene (see Data and Methods for details). For this dataset, we expect the perturbation to the global gene expressions to be smaller than when comparing two different types of cells. Indeed, our peak of histogram normalization procedure resulted in a median of base-2 logarithm of expression difference ratio between the wild type and knockout gene of 0.014, which is to be compared to 0.025 for the median under the RPKM normalization procedure. This showed that peak normalization was comparable to and perhaps slightly better than RPKM normalization.

Normalization is performed according to the assumption that most of the genes do not change expression in the two experimental conditions. Although this convenient assumption is probably true in most cases, it has no ironclad biological justification.

### Binomial distribution fit the variance from the same library but not for different libraries

**Figure 2:**
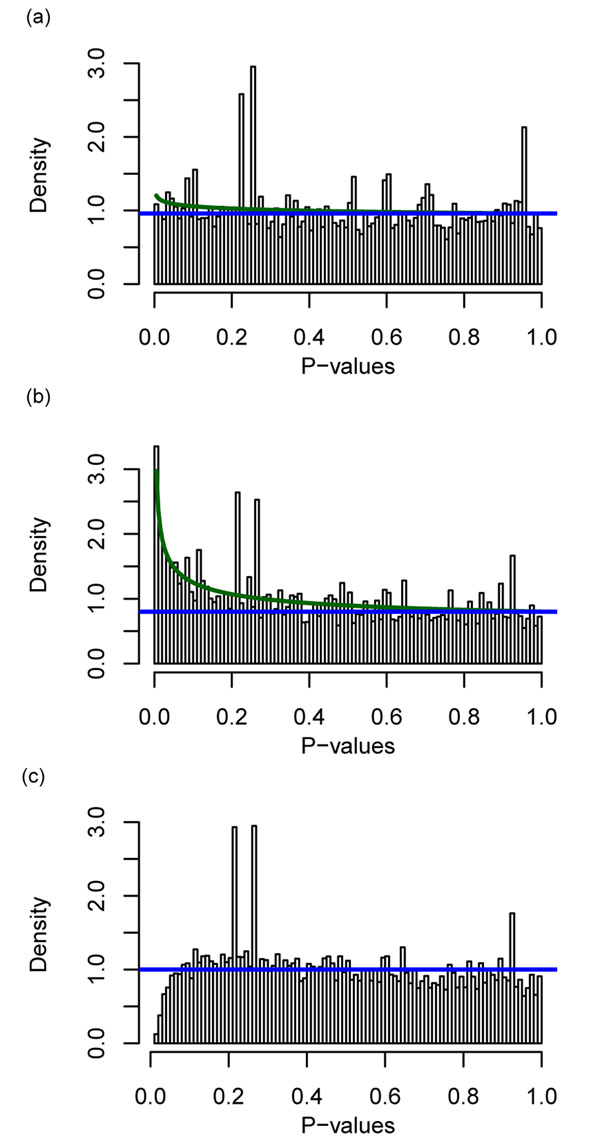
**Histogram of *p*-values of gene expression differences from duplicate experiments on the same biological sample.** (a) Duplicate experiments were from the same DNA library sequenced in different lanes. *p*-values were calculated from binomial distribution. (Two datasets compared: Bullard SRR037457 vs SRR037458.) (b) When binomial distribution is applied to the same biological sample prepared in two different libraries, more genes had small probability than expected, which erroneously predicted the existence of significantly differentially expressed genes when there should not be any. (Two datasets compared: Bullard SRR037467 vs SRR037471.) (c) When the same two libraries are compared using beta-binomial distribution, there is no longer high density at small *p*-value. Peak of proportion normalization was used in these calculations. These histograms were drawn using R package Bum-class [27].

We empirically studied errors in RNA-Seq experiments by examining the variance from replicated measurements. We first examined the fluctuation in reads mapped to a gene from duplicate experiments based on the same biological sample. The *p-*values of the differences were computed according to a binomial distribution by comparing to a neutral ratio *p_n_* as determined by peak normalization. For the same sample and the same library preparation sequenced in different lanes of the Illumina sequencer, the histogram of the *p-*value is flat (Figure 2a). This indicates that the errors in different lanes containing sample from the same library are consistent with the binomial distribution. In contrast, the histogram of *p-*values according to the binomial distribution for two independent library preparations showed clear overabundance of small p-values (Figure 2b). This demonstrated that the binomial distribution does not adequately describe the data—the dispersion of the random fluctuation is stronger than that given by the binomial distribution. We use the term library preparation to refer to an independent extraction of RNA, conversion to DNA and PCR amplification of DNA. Since the experiment and the control must be in separate library preparations, it is important to capture this overdispersion. The overabundance of small *p-*values for different libraries was also true when we used Fisher’s exact test (data not shown). When we used the beta-binomial distribution to compute the *p-*value for the different libraries, the histogram was flat. This shows the overdispersion is accounted for by the beta-binomial distribution. A Q-Q plot against either a binomial or beta-binomial distribution (data not shown) also indicated that the beta-binomial distribution better fitted the data.

### Errors decreased with sequencing depth

**Figure 3:**
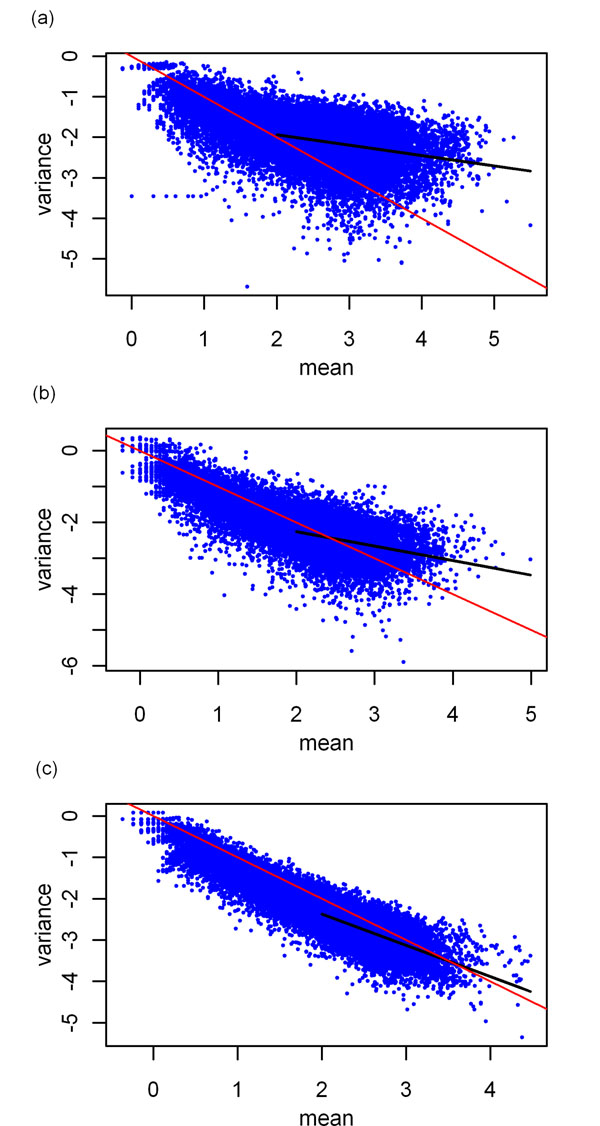
**The variance of proportion versus the mean tag counts in base-10 log scale**. The variances of proportion were computed from replicates of the same biological samples. (a) Caltech dataset; (b) Chiang dataset;(c) Bullard dataset. Each point represents a gene averaged over replicates (see Table 2 for the number of replicates for each dataset). The red line has a slope of -1. The black line is fit to the data for a mean (x-axis) larger than 2 (count greater than 100).

We first addressed the uncertainty in the RNA-Seq measurement and how uncertainty was related to the sequencing depth empirically from repeated measurements. Specifically, from replicates of the biological sample, we calculated the standard deviation of the proportion. If the proportion satisfied the binomial distribution, we expected , where *n_i_* and *m_i_* are tags mapped to gene *i* in two duplicate experiments of the sample (possibly from different libraries),  and *p_n_* is the normalization proportion. Figure 3 shows a plot of , averaged over pairs of duplicate experiments (Table 1), as a function of the mean *n_i_* + *m_i_* for the three sets of experimental data. These figures show that the variance of the proportion continued to decrease at large *n_i_* + *m_i_* and there was no sign of saturation. However, the rates of decrease with the tag counts depended on the dataset and were slower than that given by the binomial distribution.

**Table 1:** three datasets

Data Set	A		B
Caltech^a^	Normal Blood		Embryonic Stem Cells

	Rep1Gm12878CellLongpolyaBow0981x32		PairedRep1H1hescCellPapErng32aR2x75

	Rep2Gm12878CellLongpolyaBow0981x32		PairedRep2H1hescCellPapErng32aR2x75

	PairedRep1Gm12878CellLongpolyaBb12x75		PairedRep3H1hescCellPapErng32aR2x75

	PairedRep2Gm12878CellLongpolyaBb12x75		PairedRep4H1hescCellPapErng32aR2x75

Chiang^b^	Knock-out of TDP-43		Wild Type

	GSM546932_A_sorted		GSM546935_B_sorted

	GSM546933_D_sorted		GSM546936_C_sorted

	GSM546934_E_sorted		

Bullard^c^	Brain	UHR library A	UHR library B

	SRR037457	SRR037466	SRR037470

	SRR037458	SRR037467	SRR037471

		SRR037468	SRR037472

		SRR037469	

^a^ from reference [28]

^b^ from reference [22]

^c^ from reference [13]

**Table 2:** Two estimations of *γ* from three datasets

Data Set	Pairs of Experiments used in calculation	Standard Error^1^	MLE^2^
Caltech	6^a^	0.26	0.2
Chiang	3^b^	0.40	0.2
Bullard	12^c^	0.76	1.0

^1^ Obtained from slope in Figure 3

^2^ from maximizing likelihood Eq.(2)

^a^ from four libraries of same biological sample

^b^ from three knockout replicates and two wild type replicates

^c^ by comparing two different libraries having four and three replicates

### Modified beta-binomial distribution

We used a beta-binomial distribution to describe the overdispersion in the data, as shown in Figure 2b. However, in the beta-binomial distribution, the standard error approaches a constant as the mean tag counts become very large, whereas empirically, the standard error follows a decreasing trend at large tag counts (Figure 3). We therefore made the following assumption about the form of the *θ* parameter in the beta-binomial distribution (see Method for details). Let *n_i_* and *m_i_* be the number of tags mapped to gene *i.* We make *θ_i_* depend explicitly on the tag counts.(1)

Under this assumption, for 0 <*γ* < 1, the asymptotic form of the variance of the proportion at large tag count *N_i_* = *n_i_* + *m_i_* according to the beta-binomial distribution is . Therefore the variance of the proportion of the modified beta-binomial distribution does approach zero at large *N*, but at a slower rate than in the binomial distribution.

### Determining the parameters *γ* and *D_i_*

**Figure 4:**
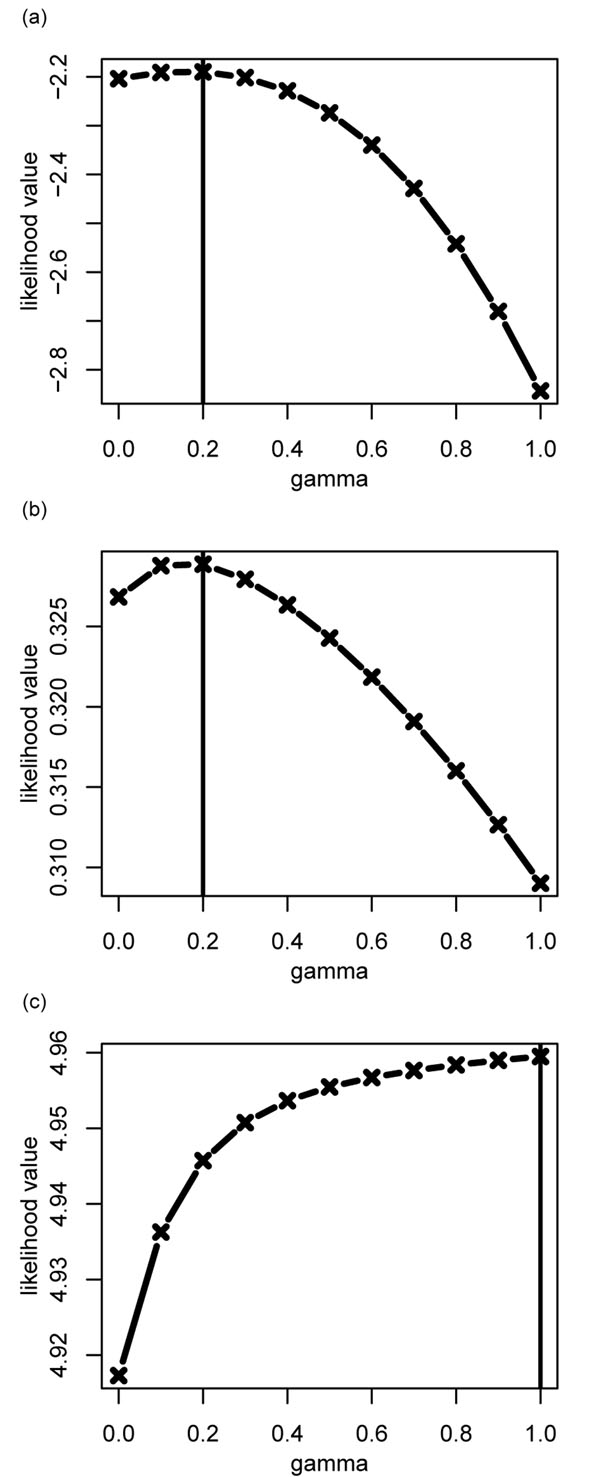
**Beta-binomial likelihood as a function of the parameter *γ***. (a) Caltech dataset; (b) Chiang dataset; (c) Bullard dataset. The vertical lines marked the position of maximum.

Although *γ* can be estimated from the slope and intercept, in the log scale of variance versus the mean tag count (Figure 3)., it required multiple experiments and had low accuracy due to data scattering. For a better estimation of the parameters *γ* and *D_i_* in Eq.(1)), we used maximum-likelihood estimation (MLE). In this approach, the likelihood was derived from the beta-binomial distribution of tag counts *n_i_* and *m_i_* for gene *i*, and summed over all the genes and over all the pairs of duplicate experiments. The overdispersion parameters *θ_i_* were given by Eq.(1) and the parameter *γ* and parameters *D_i_* for each gene were chosen to maximize the likelihood. The plots in (Figure 4). were obtained by performing a full optimization of likelihood Eq.(eq:likelihood) (see Data and Methods) with respect to *D_i_* for each *γ*, and plotting the optimized likelihood values against *γ*. Table 2 compares the *γ* from two estimates. The estimated *γ* depended on the data. We computed *γ* for three sets of data. The values ranged from 0.2 to 1.0 (Figure 4). These estimates were consistent with those from the standard error (Figure 3).

### Comparison of beta-binomial and binomial distributions

**Figure 5:**
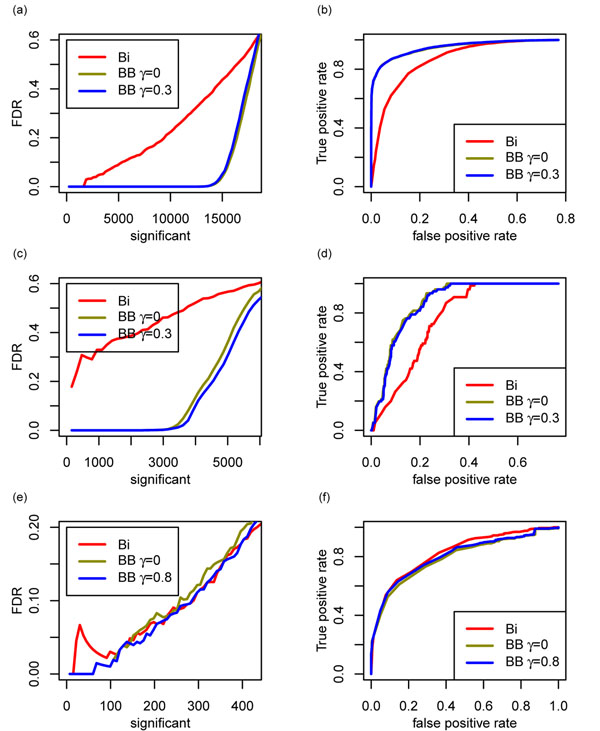
**False discovery rate (FDR) and receiver operating characteristic (ROC) for three data sets.** (a) and (b) Caltech dataset; (c) and (d) Chiang dataset; (e) and (f) Bullard dataset. Three panels on the left indicate the FDR. FDR (on y-axis) is plotted against the number of most significantly differentially expressed genes (on x-axis). Three panels on the right indicate the ROC. Bi denotes binomial distribution; BB denotes beta-binomial distribution. The line for BB *γ* = 0 was obtained by setting *γ* = 0 and optimizing *D_i_*. It corresponds to the normal beta-binomial distribution. In (b), the line for BB *γ* = 0 overlap with the line for BB *γ* = 0.3.

Figure 5 shows a comparison of the false discovery rates (FDR) [23] and receiver operating characteristics (ROC) [24] for genes deemed to be differentially expressed by the binomial and beta-binomial distributions. For the Bullard dataset, the results were comparable for the two distributions. For the Caltech and Chiang datasets, the beta-binomial distribution was superior (for dataset details, see Data and Methods).

**Figure 6:**
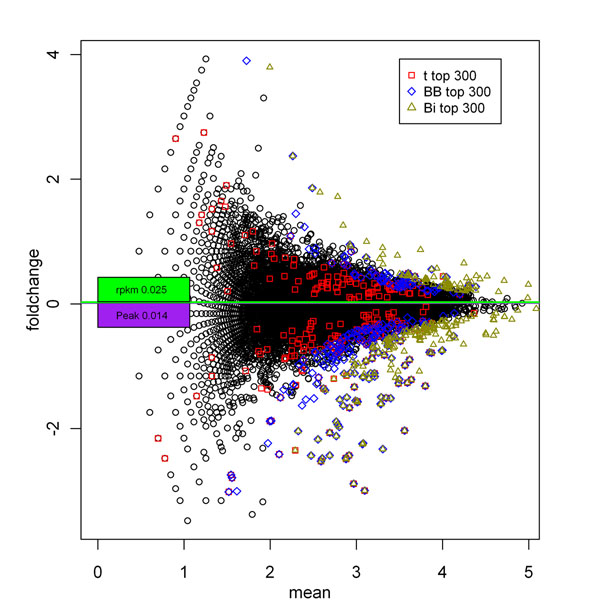
**Gene expression fold change in the TDP-43 deletion vs wild type genes (Chiang dataset).** Gene expression fold change is plotted against the average tag counts (x-axis in base-10 log; y-axis in base-2 log). The 300 most significantly differentially expressed genes by *p-*value are depicted by squares (t-test), diamonds (beta-binomial distribution), and triangles (binomial distribution). Black circles represent genes not among the top 300 in any methods. The green and purple boxes and lines indicate the median for RPKM and peak of proportional normalization. The data were from the average of three deletion and two wild type experiments.

**Figure 7:**
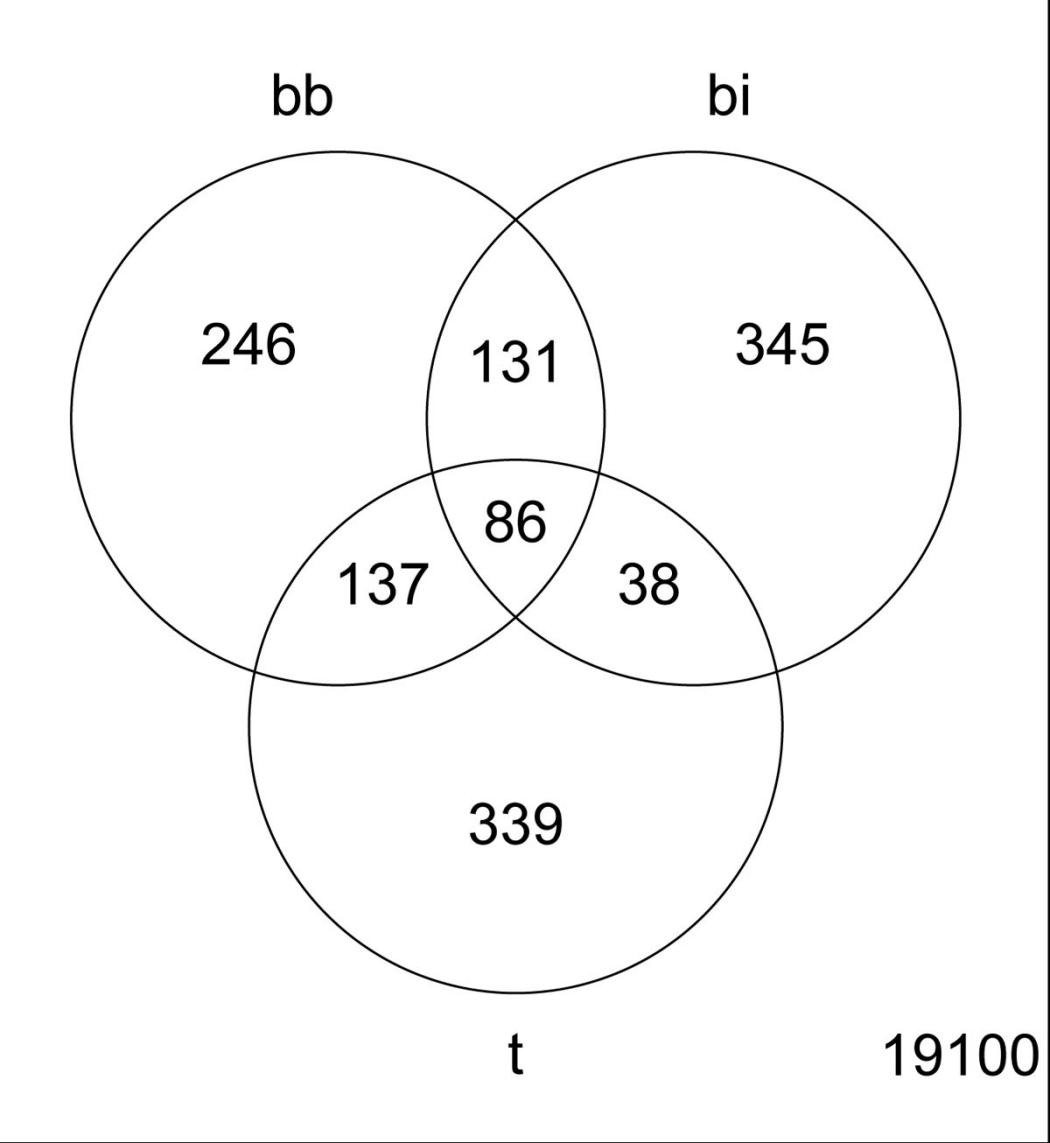
**Venn Diagram comparison.** The overlap of top 300 genes identified by beta-binomial (bb) binomial (bi), and the t-test (t) shows in Venn Diagram. The number in lower right of the rectangle indicates the total number of transcripts detected.

We took the top 300 genes deemed most significantly differentially expressed by a t-test, and by binomial and beta-binomial distributions, and overlaid them in a plot of the fold change versus the average tag counts (see Figure 6 and Figure 7). We note that the genes identified as significantly differentially expressed by the binomial distribution tended to have large tag counts; whereas many genes identified as significantly differentially expressed from the t-test had small tag counts. Some genes identified as significantly differentially expressed by the binomial distribution (marked by a triangle only) were not identified as significantly differentially expressed by the beta-binomial distribution, even though they had higher fold changes than other genes at similar tag counts. The large fluctuations of these genes are evident because they were also not called significantly differentially expressed by the t-test.

## Conclusions

We have investigated the error of RNA-Seq gene expression from repeated measurements. We have shown that the sequence reads from the same biological sample sequenced in different lanes follows a binomial distribution and that the library preparation steps prior to sequencing introduced larger variations from repeated experiments of the same biological specimen. We showed that the accuracy from repeated measurement improved with the sequencing depth. However the improvement with the tag counts was generally slower than predicted by the binomial distribution. We used a beta-binomial distribution to fit the inter-library overdispersion and introduced a parameterization of the overdispersion parameter that is consistent with the intuition that measurement accuracy should increase with the sequencing depth. We optimized the overdispersion parameters using maximum-likelihood estimation. We demonstrated better performance in lower FDR using our modified beta-binomial model.

Using the proportion of counts to estimate the gene expression difference has advantages over the RPKM expression. It has been shown recently that, in contrary to a naive presumption, the number of tags mapped to different positions in the same gene are highly non-uniform [18]. The Poisson rate at different positions can fluctuate by a few hundred fold. However, the pattern of the variable rates along the position of the gene is highly reproducible, even when comparing experiments performed on different tissues. And such rates depend on the nucleotide composition of the local sequences. The main contribution of the variable Poisson rate is that it can be attributed to the hexamer primer in converting RNA to DNA [19]. These data suggest that uneven PCR amplification could be the cause of the overdispersion that was observed. In estimating gene expression differences using a proportion, the highly variable Poisson rates do not need to be estimated. Such rates only enter the process indirectly through the dispersion. Not having to estimate the highly variable Poisson rates is therefore advantageous.

When the value of *γ* in Eq.(1) is zero, our model reverses back to the beta-binomial distribution. Interestingly, the *γ* values estimated in the different datasets were not the same. This phenomenon is similar to the GC bias in sequencing data, which also depends on the experiments [25]. Therefore *γ* may be influenced by the experimental protocol that is used. The Caltech and Chiang datasets had similar values of *γ*. In these two datasets, the *D_i_* values are similar for the same gene (data not shown). This is quite consistent with the previous finding [18] that the variability of the Poisson rates is similar in different experiments. It would be interesting to study how *D_i_* may depend on the DNA sequences of the gene. Another parametrization of overdispersion is by position within a gene using Eq.(1). These possibilities will be explored in the future.

## Methods

### Peak of proportion histogram normalization

The normalization procedure using the peak of the histogram of proportion assumes that most genes remain unchanged in the two conditions being compared. In this normalization procedure, we fitted the highest peak in the histogram of proportion to a beta function. The maximum of the beta function determines the normalization proportion *p_n_.*

In RPKM normalization, we first count the total number of tags mapped to any gene in the RNA-Seq experiment. The number of tags mapped to a particular gene is divided by the total number of tags sequenced (the unit is millions of tags), and then divided by the number of nucleotides in the gene (the unit is thousands).

### Datasets used

The three datasets we used are listed in Table 1.

The *Chiang dataset* consisted of five independent libraries of the deleted TDP-43 gene in the mouse. The data were derived from three independent clones of TDP-43 knockout embryonic stem (ES) cells and two independent clones of control ES cells. Raw reads were mapped to the University of California Santa Cruz mm9 genome library by efficient large-scale alignment of nucleotide databases. One gene deletion is an ideal case for testing normalization procedures with the assumption that most genes do not change.

The *Caltech dataset* consisted of two cells lines: GM12878 (normal blood) and H1hESC (embryonic stem cells), each with four libraries made independently from the same biological sample. The process involved raw Illumina reads on 2x75 datasets (RawData files on the download page, fasta format), which were run through Bowtie, version 0.9.8.1, with up to 2 mismatches. The resulting mappings were stored (RawData2 files, Bowtie format) for up to ten matches per read to the genome, spiked controls and UCSC knownGene splice junctions.

The *Bullard dataset* consisted of human brain reference RNA and human universal reference RNA as two library preparations. We used Bowtie, version 0.12.7, to align the reads to the genome (H. sapiens, NCBI 37.1 assembly). The Bowtie command we used to implement this mapping strategy was ./bowtie -a -v 2 -t -m 1 --best -strata h_sapiens_37_asm.

### Maximum-likelihood estimation (MLE)

Let *n_ip_* and *m_ip_* be the tags mapped to the *i*-th gene and *p*-pair of experiment and control, respectively. The likelihood function according the beta-binomial distribution is

where α*_ip_* and *β_ip_* are two parameters of the beta-binomial distribution. This is equivalent to using instead the following parameters , and . It can be shown analytically that the proportion that maximizes the likelihood function is given by . We will further assume that *θ_ip_* is independent of of *p*; we use Eq.(1) to reparameterize *θ_i_* in terms of parameters *D_i_* and . The parameters were determined by maximizing the likelihood(2)

### Likelihood ratio test

According to the likelihood ratio test,  follows a *Χ*^2^ distribution, where *p_i_* is the proportion for gene *i* and *p_n_* is the normalized proportion corresponding to no change in gene expression. This is the most convenient way to compute the *p-*value.

### FDR and ROC

To determine the false discovery rate (FDR), we assumed that any gene deemed to be significantly differentially expressed at a given *p*-value were false when comparing two replicates sequenced from the same biological sample. We computed the FDR by dividing the number of falsely discovered genes at a given *p-*value with the number of significantly differentially expressed genes, comparing the sample to the control at the same *p-*value.

To determine the receiver operating characteristic (ROC), we first established a gold standard. Approximately one thousand genes in the Bullard dataset were previously assayed by RT-PCR in four independent experiments [26]. Differentially expressed genes were determined by t-test by Bullard et al. [13]. We used their results to draw an ROC curve when comparing the binomial and beta-binomial distributions for the Bullard dataset. For the Caltech and Chiang datasets, we assumed that the t-test provided a gold standard. In order to reduce errors for small tag counts, we required a gene to have more than 20 mapped tags. For the Caltech data, the Benjamini & Hochberg adjustment was applied to the *p*-value calculated by the t-test, using a cutoff of 0.05 [23]. We could not use the FDR *p-*value adjustment on the Bullard dataset, as much fewer genes had differential expression levels detected from the wild/knockout samples. Therefore, we applied a cutoff of 0.05 to the *p-*value from the t-test and required a fold change larger than two.

### Computing the fold change

We related the fold change in the gene expression level FC*_i_* to the optimized ratio *p_i_* and obtained, by definition, . This ratio has to be calibrated against the normalization of the entire experiment. We defined *p_n_* as no change. Therefore , where *p_n_* is the normalized ratio as determined over the entire dataset. Infinite values of FC*_i_* can be avoided by adding a pseudo-count to *n_i_* and *m_i_* so that 0 <*p_i_* < 1.

## Competing interests

The authors declare that they have no competing interests.

## Authors' contributions

SL and GC designed the studies. GC wrote perl and R program and performed data analysis and modeling. HL and YL assisted in data analysis and modeling. SL, GC, XH, JL, PM, YJ developed statistical model. SL and GC wrote the manuscript.
